# Botulinum Toxin-A, Generating a Hypothesis for Orofacial Pain Therapy

**DOI:** 10.3390/toxins17080389

**Published:** 2025-08-04

**Authors:** Yair Sharav, Rafael Benoliel, Yaron Haviv

**Affiliations:** 1 Department of Oral Medicine, Sedation and Imaging, Hadassah Medical Center, Faculty of Dental Medicine, Hebrew University of Jerusalem, Jerusalem 91120, Israel; sharavy@mail.huji.ac.il; 2 Rutgers School of Dental Medicine, Newark, NJ 07103, USA; benolira@sdm.rutgers.edu

**Keywords:** botulinum toxin, BoNT-A, trigeminal neuralgia, temporomandibular disorder, neuropathic pain, migraine, orofacial pain, neuromodulation, one-time pain prophylaxis

## Abstract

Orofacial pain encompasses a spectrum of disorders ranging from musculoskeletal disorders, such as myofascial pain, and temporomandibular disorders to neuropathic situations, such as trigeminal neuralgia and painful post-traumatic trigeminal neuropathy, and neurovascular pain such as orofacial migraine and cluster orofacial pain. Each require tailored prophylactic pharmacotherapy, such as carbamazepine, gabapentin, pregabalin, amitriptyline, metoprolol, and topiramate. Yet a substantial subset of patients remains refractory. Botulinum toxin type A (BoNT-A) has demonstrated growing efficacy in the treatment of multiple forms of orofacial pain, which covers the whole range of these disorders. We describe the analgesic properties of BoNT-A for each of the three following orofacial pain disorders: neuropathic, myofascial, and neurovascular. Then, we conclude with a section on the neuromodulatory mechanisms of BoNT-A. This lays the basis for the generation of a hypothesis for the segmental therapeutic action of BoNT-A on the whole range of orofacial pain disorders. In addition, the advantage of BoNT-A for providing a safe sustained effect after a single application for chronic pain prophylaxis is discussed, as opposed to the daily use of current conventional prophylactic medications. Finally, we summarize the clinical applications of BoNT-A for chronic orofacial pain therapy.

## 1. Introduction

Orofacial pain encompasses a spectrum of disorders ranging from musculoskeletal disorders such as myofascial pain, and temporomandibular disorders to neuropathic situations such as trigeminal neuralgia and painful post-traumatic trigeminal neuropathy, and neurovascular pain such as orofacial migraine and cluster orofacial pain [1]. These entities present with distinct symptomatic profiles associated with various underlying pathophysiological mechanisms. Each disorder necessitates tailored prophylactic pharmacotherapy, such as carbamazepine for trigeminal neuralgia, gabapentin and pregabalin for neuropathic pain, amitriptyline and gabapentin for myofascial orofacial pain, and metoprolol and topiramate for orofacial migraine. Despite various pharmacologic and interventional options, a substantial subset of patients remains refractory. Botulinum toxin type A (BoNT-A), a neurotoxin traditionally used for dystonia and esthetic applications, has increasingly been shown to have efficacy in the management of multiple forms of orofacial pain. Recent evidence has identified BoNT-A as a promising, localized therapeutic strategy in the treatment of orofacial pain that covers the whole range of these disorders [2,3,4,5,6,7,8].

The underlying mechanisms enable BoNT-A to provide successful treatment encompassing the whole spectrum of trigeminal pain disorders, not obtained by most other pharmacotherapeutic agents. This may be due to trigeminal segmental activity enabling neural block in segmental treatment [9]. Furthermore, in comparison with conventional prophylactic analgesics that need to be administered daily, a great advantage of BoNT-A is that it has a sustained long-term effect after a single application with mild side effects [10].

Despite their diverse etiologies, neuropathic, myofascial, and neurovascular orofacial pain conditions may share overlapping peripheral and central mechanisms. In this paper, we propose a unified hypothesis: BoNT-A exerts analgesic effects across the spectrum of orofacial pain disorders via dual mechanisms, segmental neural blockade and retrograde axonal transport leading to central neuromodulation. We aim to conceptually integrate current clinical and preclinical findings into a cohesive mechanistic framework that explains the broad efficacy of BoNT-A, and to suggest new directions for targeted, condition-spanning therapies.

Subsequently, we describe the analgesic effects of BoNT-A for each of the three orofacial chronic pain disorders: neuropathic, myofascial, and neurovascular. This is followed by a section on the neuromodulator pharmacotherapeutic mechanism of BoNT-A, indicating possible explanations for the therapeutic action of BoNT-A on the whole spectrum of orofacial pain disorders, and leading to the generation of a hypothesis for orofacial pain therapy using BoNT-A. We conclude with the clinical applications of BoNT-A therapy for orofacial pain.

## 2. BoNT-A Effect on Neuropathic Pain

### 2.1. BoNT-A Effect on Trigeminal Neuralgia

Trigeminal neuralgia (TN) is a unilateral facial pain with pronounced physical signs and symptoms and is debilitating, significantly affecting quality of life [11,12]. The pain is excruciating, short-lasting, usually described as electrical or sharp and is “triggered” by light touch in the affected area. Classical TN (CTN) is defined as occurring with “demonstration on MRI or during surgery of neurovascular compression (not a simple contact), with morphological changes in the trigeminal nerve root” [13]. Symptomatic TN is caused by underlying pathology or disease that cause demyelination and/or compression. Idiopathic TN (ITN) is defined as “Trigeminal neuralgia with neither electrophysiological tests nor MRI showing significant abnormalities [13]”. The striking issue with ITN is that there is no compression resulting from a neurovascular contact and no other known causative factors. Further research is needed to elucidate pain mechanisms. Carbamazepine is highly efficacious in TN [14] and is usually the first drug tested [15]. The number needed to treat (NNT) for any pain relief for carbamazepine in CTN is 1.9 and for significant effectiveness is 2.6 [16]. Recent studies established that, over a two year follow-up, TN patients do not generally become refractory to medication [14]. BoNT-A has been shown to be a possible choice of treatment for TN, and may be an efficient, safe, and novel strategy. It is especially useful in patients unable to use carbamazepine due to adverse effects or allergic reactions. The use of carbamazepine may be accompanied by adverse effects such as dizziness [17]. Carbamazepine and oxcarbazepine are the most used and effective treatment options for TN; almost 90% of patients have initial pain relief, but 40% have significant adverse events, determining withdrawal of the drug [18]. BoNT-A therapy is simple and can be performed as an outpatient procedure without anesthesia, and the toxin does not cause regional sensory loss or dysesthesia [19].

In a randomized, double-blind, placebo-controlled study, 42 TN patients were allocated into two groups, namely BoNT-A (75 U/1.5 mL; n = 22) (100 U of Clostridium botulinum type A neurotoxin complex, 5 mg gelatin, 25 mg dextran, and25 mg saccharose was obtained from Lanzhou Biological Products Institute, China. The content of each vial was diluted in 2 mL saline solution (0.9%) as recommended by the manufacturer), or saline (1.5 mL; n = 20) injection intradermally and/or mucosal where pain was experienced [20]. The primary endpoints were reduction in pain severity and pain attack frequency per day at week 12. More responders were significantly present in the BoNT-A group (68.18%) than in the placebo group (15.00%). BoNT-A was well tolerated, with few treatment-related adverse events [20].

A randomized, double-blinded, placebo-control study was carried out on 20 patients with intractable TN defined as failing to experience at least 50% pain reduction during the last 3 months, despite the use of appropriate drugs and dosages [21]. Patients were randomly allocated into two groups with 10 patient each, and received a one-time subcutaneous injection of BoNT-A (Botox^®^), 100 U Botox in 2 mL preservative-free normal saline, resulting in a concentration of 5 units/0.1 mL) or saline in the area of pain [21]. Pain reduction, measured with a visual analog scale (VAS), relative to a baseline at the 12-week endpoint was significantly reduced by 6.5 in the BoNT-A group (*p* < 0.0001) compared with a non-significant decrease of 0.3 for the placebo. There was a significant decrease in the number of acute medications and an increase in the quality of life (QoL) scale in the BoNT-A group [21].

The efficacy, safety, and tolerability of either 1 mL 0.9% saline containing 50 U of BoNT-A (BOTOX [BTX]) or 1 mL of 0.9% saline (placebo) injected subcutaneously in the affected area were evaluated in patients with TN [22]. Twenty subjects were administered BoNT-A, and 16 subjects received the placebo. Two months after the intervention, a larger reduction in mean VAS values in subjects treated with BoNT-A and those who received the placebo was observed (VAS 4.9 vs. 6.63, *p* = 0.07). Three months after the injection, significant differences were observed in the average VAS score for subjects treated with BoNT-A and those treated with the placebo (VAS 4.75 vs. 6.94, respectively; *p* = 0.01) [22].

In a double-blind, placebo-controlled study, 80 trigeminal neuralgia (TN) patients were randomized into the following groups: placebo (n = 26); BoNT-A 25 U (n = 26); and BoNT-A 75 U (n = 28) [23]. The BoNT-A BTX-A (100 U of Clostridium botulinum type A neurotoxin complex, 5 mg gelatin, 25 mg dextran, and 25 mg saccharose), obtained from Lanzhou Biological Products Institute, Lanzhou, China, or the same volume of isotonic saline were applied at 20 points, (0.05 mL) per point, between the epidermis and dermis of the skin where pain was experienced according to the patient’s description. The injections were conducted submucosally in the oral mucosa if the pain involved the oral mucosa. During the procedure, injection in deeper structures such as the muscles was avoided to prevent undesirable effects. The response rates of the 25 U group (70.4%) and the 75 U group (86.2%) were significantly higher than the placebo group (32.1%) at week 8 (*p* < 0.017). There was no significant difference between the 25 U and 75 U groups (*p* > 0.05) [23].

An open-label trial was conducted in 100 patients with TN, divided into two groups in order to compare a single injection vs. repeated injections of BoNT-A response to treatment. Response to treatment was determined as the percentage of patients with a ≥50% reduction in mean pain score from baseline to endpoint. During a 5-month follow-up, response rates were in the range of 65–90% of patients. Results indicated no significant difference between the single- and repeated-dose groups (*p* > 0.05). However, the duration of efficacy in the single-dose group was significantly longer than that of the repeated-dose group (*p* = 0.032) [24]. It was concluded that repeated dosing has no advantage over single dosing of BoNT-A for TN [24].

In a systematic review of TN, BoNT-A injection compared to saline injection demonstrated the superiority of BoNT-A in VAS reduction (68%) compared to saline (21.6%), with a decrease in attack frequency (85% vs. 15.9%) [25]. No differences between dosages of BoNT-A were found. Maximum efficacy was noticed between 6 weeks and 3 months after the procedure. Side effects were mostly facial asymmetry after injection, and also headaches, or hematoma, which disappeared in one week [25].

In conclusion, BoNT-A is a viable addition to the treatment options of patients with TN, especially when traditional pharmacotherapy such as carbamazepine is ineffective or contraindicated because of adverse effects or allergic reaction. There is, however, a need to establish proper dosing [23,25]. Of special interest is the efficacy of a single prophylactic application that is equal, or may even be superior, to multiple injections [24]. Therefore, BoNT-A has the advantage of a one-time prophylactic single application rather than the daily consumption of medications such as carbamazepine. BoNT-A is well tolerated with minimal adverse effects.

### 2.2. BoNT-A Effect on Peripheral Painful Traumatic Trigeminal Neuropathy (PTTN)

Painful traumatic trigeminal neuropathy (PTTN) may occur following major craniofacial or oral trauma [26,27], as well as relatively minor dental interventions [28]. Local anesthetic injections may induce nerve injury secondary to physical trauma by the needle or by chemical insult from the anesthetic solution [29,30]. A common neuronal complication following implant insertion is damage to adjacent nerves, altered sensory perception, and possibly pain [31,32]. Persistent pain after successful endodontics was found in 3–13% of cases, and chronic neuropathic pain may reach 5% of cases of surgical endodontics [28,33,34].

In contrast to the traditional 50% pain reduction used to describe pain reduction of clinical significance, research studies have shown that about a 30% reduction represents meaningful pain relief for neuropathic pain patients [35]. Therapy of neuropathic pain with any one of the established drug groups (antidepressants, anticonvulsants) leads to improved quality of life, sleep, and mood. However, pain intensity is reduced by 20–40% in only a subset of responders and is usually accompanied by significant side effects, particularly at the higher doses often required in neuropathic pain [36,37,38]. The poor response of PTTN to therapy calls for a great need for additional pharmacotherapeutic agents, and the use of BoNT-A has gained increased attention and importance in the management of peripheral neuropathic pain.

In a retrospective case series of seven patients, five of the patients with PTTN experienced significant pain reduction after BoNT-A injection. Five of the seven patients received intraoral injections in the gingival vestibule or mucosa, while the remaining two received extraoral injections in the masseter and temporal muscle areas. Three types of BTX-A were used: Meditoxin (Medytox Inc., Seoul, Republic of Korea), Innotox, and Dysport (Ipsen Biopharm Ltd., Slough, UK), and the number of injections ranged from at least once to a maximum of three times every 3 months [3]. These PTTN patients were previously administered a variety of medications that were discontinued due to being ineffective or due to side effects. In the other two patients, with atypical orofacial pain who complained of persistent pain without any causative factors, the pain was unaffected by injection of BoNT-A [3].

The management of PTTN was addressed in a topical review, indicating that data is based on case reports. It concludes that, although scarce, the data is regarding the use of BoNT-A and clearly underlines the need for further randomized control trials (RCTs) on its use for PTTN treatment [39].

A detailed case report of a patient with PTTN was presented [40]. There was a 3-year history of chronic intractable spontaneous and evoked pain due to inferior alveolar and buccal nerve injury following mandibular third molar extraction. The case had been successfully managed with BoNT-A (50 IU in 1 mL saline, BotoxVR; Allergan, Dublin, Ireland) injection. BoNT-A infiltrated intraorally to the posteroinferior aspect of the mental nerve bundle (which innervates the chin region) in order to avoid labial paralysis [40]. At the 3-month follow-up, after successful management, the pain had progressively returned to its initial stage, and the patient sought another injection. A repeat injection was performed at that time with similar efficacy [40].

A systematic review by Val et al. [4] on BoNT-A effectiveness in orofacial neuropathic pain disorders concluded that although there is evidence, there is a lack of stringent studies.

A recent update addresses the effect of BoNT-A on a variety of neuropathic pains, including painful diabetic neuropathy (PDN), post-traumatic painful neuropathy/neuralgia (PTN), post-herpetic neuralgia (PHN), and occipital neuralgia (ON) [5]. The authors concluded that the current levels of efficacy for these conditions could be designated as following: PDN: B (probably effective, two class II study), PTN: A (effective, two class I studies); PHN: A (effective, two class I studies); and ON: (undetermined due to lack of blinded investigations). Due to the small number of patients in these studies, proof of efficacy requires controlled and blinded studies in larger cohorts of patients with longer follow-up [5].

Preclinical studies in rats, using the infraorbital nerve chronic constriction injury, demonstrate the antinociceptive effect of BoNT-A [41,42]. The observed bilateral effects of BoNT-A (Botox, Allergan, Inc., Irvine, CA, USA) and dependence on retrograde axonal transport suggest a central site of action [41]. Additionally, BoNT-A (Hengli, Lanzhou, China) exerted antinociceptive effects lowering the expression of vanilloid receptors in the subnucleus caudalis of the trigeminal nucleus [42]. In another preclinical study in rats, mechanical allodynia was induced by injury of the inferior alveolar nerve by dental implants [43]. The mechanical allodynia was attenuated by BoNT-A (Botulax; Hugel, Inc., Chuncheon-si, Gangwon-do, Republic of Korea), which significantly inhibited the upregulation of Nav 1.7 expression in the trigeminal ganglion [43]. Recent studies carried out in animal models indicate that remote or local induction of facial neuropathic pain could be alleviated by locally administered BoNT-A [44]. Furthermore, unilateral peripheral administration of BoNT-A to the rat whisker pad attenuated cisplatin-induced bilateral facial neuropathic pain. Moreover, contralateral peripheral administration of BoNT/A attenuated neuropathy-induced behavior caused by infraorbital nerve constriction. These findings point to the involvement of axonal transport in the therapeutic effects of facial neuropathic pain by peripherally administered BoNT-A in the orofacial region [44].

In conclusion, BoNT-A shows promising results in the management of PTTN, yet data is based on small trials and case reports. Most studies were preclinical and based on observations in mice and rats. However, these animal studies demonstrated the central antinociceptive effect of BoNT-A on peripheral neuropathic pain. There is therefore a need for further human studies in large cohorts with longer follow-ups.

## 3. BoNT-A Effect on Chronic Myofascial Orofacial Pain

Temporomandibular disorders (TMDs) affect approximately 5–12% of the population and represent a major source of chronic non-dental orofacial pain [45]. Chronic myofascial orofacial pain (MOP), a subtype of TMD is characterized by persistent regional or referred pain, reduced mandibular function, and impaired quality of life. Pain on palpation is usually present in the ipsilateral masticatory muscles; the masseter is the most commonly involved muscle (>60%), followed by the medial pterygoid and temporalis muscles in about 40–50% of cases [46]. Typically, there are localized tender sites and trigger points in muscle, tendon, or fascia [47,48]. However, the importance of trigger points remains controversial and they may be an epiphenomenon of deep tissue pain [49].

Standard treatment approaches include physical therapy, pharmacotherapy, behavioral interventions, and occlusal appliances. Management with a variety of conservative methods consistently results in high (75–90%) success rates [50,51,52,53,54]. In general, treatment is aimed at palliation and is based on clinical diagnosis; since the etiology and underlying pathology remain unclear, no treatment is curative, and 10–25% of patients are refractory to management [51].

BoNT-A has emerged as a potential alternative or adjunctive treatment for patients with refractory TMD. While originally developed for spastic neuromuscular conditions, its analgesic and neuromodulator properties have expanded its use to various chronic pain disorders, including TMD. BoNT-A blocks acetylcholine release at the neuromuscular junction, inducing localized, temporary muscle relaxation. Beyond this, it also reduces the release of pain-related neurotransmitters, substance P, CGRP, and glutamate, from peripheral nociceptors. BoNT-A suppresses the activity of TRPV1 receptors and may retrogradely modulate central pain processing [55,56]. These combined effects reduce the hyperactivity of masticatory muscles and interrupt the cycle of peripheral and central sensitization [6,55,57,58]. One cannot, therefore, entirely dismiss the notion that chronic myofascial temporomandibular pain is associated with neural abnormalities in the trigeminal and limbic systems [56], which supports the rationale, as exercised by BoNT-A mechanisms, of the role of therapy by central modulation.

Early randomized trials by von Lindern et al. [59] studied 90 patients with chronic masticatory muscle pain; 60 received BoNT-A (Botox) and 30 received saline, injected into the masseter and temporalis muscles. They demonstrated significant reductions in pain following BoNT-A injections.

Intramuscular injection of BoNT-A in the area of the greatest cross-section surface of both masseter bellies in 42 subjects with masseter muscle pain was evaluated for pain reduction [60]. Pain intensity was measured 1 week before and 24 weeks after the treatment. The results showed a significant decrease in the number of referred pain episodes including a decrease in pain in the temporal region bilaterally, a reduction in analgesic drugs intake, and a decrease in reported VAS and NRS values after injections [60].

However, BoNT-A treatment of chronic myofascial orofacial pain has yielded inconsistent therapeutic outcomes, [7,61,62,63]. In a multicenter study of 21 patients with persistent myofascial TMD pain, the number of patients who received a 30% pain reduction was not significantly larger for BoNT-A than after saline at any follow-up visit. These results do not indicate a clinically relevant effect of BoNT-A (Botox; Allergan Norden AB, Upplands Väsby, Sweden) in patients with persistent myofascial TMD pain [64]. Moreover, when looking at studies in the management of chronic muscle pain in the neck or other regions, there is insufficient evidence to support the use of BoNT-A [7,65,66,67]. Due to the considerable variations in study methods and inconsistent results [7], the role of BoNT-A in the management of chronic muscle pain needs further research. A recent systematic review concluded that analysis of the studies has shown that low doses of botulinum toxin are effective in the treatment of refractory myofascial pain associated with temporomandibular disorders, but these studies presented medium- to low-certainty evidence [68].

Clinical experience suggests that injections of local anesthetics into “trigger points” induce pain relief beyond the effect of the anesthetic agent [69]. In MOP patients, bupivacaine (0.5%) is equi-efficacious to 25 units of BoNT-A mixed with 1/2 cc normal saline per trigger point) in the relief of pain, and cost effectiveness would suggest the former’s preferential use [70]. Furthermore, BoNT-A injections have not been found to be superior to placebo injections in MOP patients [64,71]. A recent systematic review suggested that there is good evidence to support the use of BoNT-A injections for the treatment of masseter hypertrophy, but the evidence for MOP is equivocal [7].

However, in a recent randomized, double-blind, placebo-controlled pilot study, Kim et al. [2] studied 21 patients with myogenous TMD randomly assigned to two groups receiving either BoNT-A (Botulax^®^, Hugel Inc., Chuncheon, Republic of Korea) or saline into the sites showing tenderness after palpation of muscles, including the masseter and temporalis as well as three neck muscles. Patients were evaluated at pre-injection and 4, 8, and 12 weeks after the injection. The intensity of orofacial pain and headache, and number of tender points were significantly reduced in the BoNT-A group, but no difference was observed in maximum mouth opening [2]. The long-term pain intensity reduction of 12 weeks achieved by BoNT-A is beyond the time span usually shown by local anesthetics, therefore indicating a specific BoNT-A analgesic mechanism. The effect of BoNT-A persists for months after its single use; apparently, the most important factor regulating the longevity of its action is the ability of BoNT-A protease to avoid cellular degradation in the cell cytoplasm for a long period [10].

In conclusion, MOP, a subtype of TMD, demonstrated significant reductions in pain and improved jaw function following BoNT-A injections into the masseter and temporalis muscles. However, BoNT-A treatment of chronic myofascial orofacial pain has yielded inconsistent therapeutic outcomes. However, the effect of BoNT-A, when successful, that persists for months after a single use, cannot be ignored. The evidence of clinical improvement suggests that BoNT-A’s mechanisms extend beyond local cholinergic blockade, involving both attenuation of peripheral nociceptive input and modulation of segmental spinal trigeminal circuits, supporting its possible role as a dual-level neuromodulator within the trigeminal system [56].

## 4. BoNT-A Effect on Facial Migraine

The term “neurovascular” reflects the suspected etiology of headaches or facial pain. Pain is considered to result, at least partly, from an interaction between the trigeminal nociceptive afferents and the craniofacial vasculature, together termed the trigeminovascular system [72]. Migraine and its facial variants are debilitating neurovascular disorders characterized by recurrent attacks of moderate to severe craniofacial pain, often accompanied by autonomic symptoms, and impaired quality of life [1]. The recent ICOP classification [1] defined two diagnostic entities of facial migraine: orofacial migraine and neurovascular orofacial pain. While these two entities carry distinct diagnostic features [73], they respond similarly to migraine prophylactic therapy [74]. Therefore, for the sake of brevity, these two diagnostic entities will be referred to as facial migraine. Low-dose amitriptyline, propranolol, and anticonvulsant therapy have been successful prophylactic drugs in facial migraine patients [75,76,77,78].

Chronic migraine (CM), defined as headache on 15 or more days per month for more than 3 months, poses a substantial challenge to both patients and clinicians [13]. BoNT-A has emerged as a key prophylactic therapy for chronic migraine and is increasingly considered in facial migraine [79,80,81,82,83,84]. BoNT-A has been the only preventive treatment for CM that has long-term safety data in real-world settings, reporting treatment-related adverse events of up to 3 years [81]. BoNT-A should be administered according to the PREEMPT injection protocol, i.e., injecting 155 U–195 U to 31–39 sites every 12 weeks. It is recommended that patients are defined as non-responders if they have less than a 30% reduction in headache days per month during treatment with BoNT-A. However, other factors such as headache intensity, disability, and patient preferences should also be considered when evaluating response [85]. BoNT-A is FDA-approved for CM but not for episodic migraine (EM). However, in clinical practice, BoNT-A is sometimes used off-label for EM, especially in high-frequency or refractory cases. A 2018 meta-analysis showed modest efficacy in EM, though heterogeneity of studies precluded definitive conclusions [84].

In order to avoid interference with mimic muscles in the facial area, it is possible to inject BoNT-A in remote areas (e.g., forehead) in order to alleviate orofacial migraine. Furthermore, the application of BoNT-A injection between the epidermis and dermis of the skin as performed for TN is also an option to avoid muscular interference [20,23].

In conclusion, BoNT-A has emerged as a key prophylactic therapy for chronic migraine independent of location. However, there is a lack of human studies on the utilization of BoNT-A in the treatment of facial migraine, and therefore, a need for randomized, double-blind, placebo-controlled studies in large cohorts of patients with long follow-ups.

To summarize, the uses of BoNT-A in orofacial pain conditions, including its efficacy, clinical notes, and injection sites, are presented in Table 1.

## 5. Mechanism of Analgesic Effects of BoNT-A

BoNT-A is a neurotoxin that causes relaxation of skeletal muscles by blocking the release of acetylcholine at the neuromuscular junctions [86,87]. Indeed, BoNT-A’s putative success in pain management was originally attributed to its ability to block acetylcholine release. BoNT-A was thought to act only on motor nerve endings while sparing sensory nerve fibers from its effects. However, some of the earliest clinical studies with BoNT-A also reported that there were significant effects on reduction in patients’ pain including for the face [88,89,90]. Subsequently, effects of botulinum toxin on nociceptive neurons were demonstrated in preclinical studies [19,91,92]. In rat models, peripheral injection of BoNT-A has been shown to inhibit the secretion of substance P, glutamate, and calcitonin gene-related peptide (CGRP) from sensory neurons and inhibits the activity of vanilloid receptors [6,55,57,58]. It also reduces local inflammation around nerve endings [57], and inhibits sodium channels that are essential for pain transmission [93]. BoNT-A prevents synaptic transmitter release by the enzymatic cleavage of the synaptosome-associated protein 25 kDa (SNAP-25), one of the three synaptic proteins that form the heterotrimeric soluble N-ethylmaleimide-sensitive factor attachment protein receptor (SNARE) complex involved in the synaptic vesicle fusion with the presynaptic membrane [94]. Its central efficacy is supported by experimental trials in animals [44] as well as clinical studies in diabetic patients [95]. BoNT-A is retrogradely transported to the spinal cord, and inhibits the release of substance P and c-Fos expression [96,97]. A recent review also suggests that BoNT-A can accelerate nerve regeneration after nerve injury to peripheral nerves, possibly by the activation or proliferation of support cells (Schwann cells, mast cells, and macrophages), increasing angiogenesis, and the improvement in blood flow to regenerating nerves [98].

Specifically in the facial area, preclinical studies in rats, using the method of infraorbital nerve constriction injury, demonstrated the antinociceptive effect of BoNT-A on this peripheral neuropathic pain [41,42]. Bilateral effects of BoNT-A and dependence on retrograde axonal transport suggest the central site of its action [41]. Moreover, BoNT-A exerted antinociceptive effects lowering the expression of TRPA1, TRPV1, and TRPV2 in the subnucleus caudalis of the trigeminal nucleus [42]. In another preclinical study, malpositioned dental implants induced neural injury in the inferior alveolar nerve in rats, resulting in mechanical allodynia [43]. This mechanical allodynia was attenuated by BoNT-A, which significantly inhibited the upregulation of Nav 1.7 expression in the trigeminal ganglion [43]. The selectivity for certain sensory neuron populations, such as TRPV1-expressing neurons, allows selectivity for chronic prolonged pain and migraine [99]. Low local diffusion after application enables intradermal injection and provides a local effect with relatively lower injection volumes, very appropriate for the orofacial region [100].

Conceptually, this retrograde axonal transport that results in the central site of action, and the segmental activity of BoNT-A permitting neural block for segmental treatment [9], provides successful treatment for all trigeminal pain disorders which Is not obtained by most other pharmacotherapeutic agents. Furthermore, in comparison with other conventional prophylactic analgesics, which need to be consumed daily, a great advantage of BoNT-A use is the sustained effect after a single application [10]. This convergence of mechanisms, integrating peripheral suppression of neuropeptides with retrograde central modulation, underpins our generating hypothesis that BoNT-A acts as a segmental neuromodulator in migraine therapy, effectively disrupting the trigeminovascular pain pathways involved in both facial pain manifestations.

In conclusion, the convergence of BoNT-A’s segmental and central mechanisms may explain its unique ability to alleviate pain across a diverse spectrum of orofacial conditions, each traditionally managed as a distinct clinical entity. This generating hypothesis suggests that BoNT-A, through localized inhibition of peripheral nociceptive transmission and retrograde modulation of central pain processing, offers a mechanism-based, long-lasting therapeutic alternative to conventional, symptom-specific daily pharmacotherapy. Its capacity to produce sustained analgesia following a single administration, unlike most pharmacological agents, positions BoNT-A as a potentially transformative modality in chronic orofacial pain management. However, current clinical support stems largely from small trials, open-label studies, and preclinical observations. To validate this generating hypothesis and fully elucidate its translational relevance, future randomized, double-blind, placebo-controlled studies in large patient cohorts are warranted, ideally integrating molecular and neurophysiological biomarkers to correlate clinical outcomes with BoNT-A’s dual-action mechanisms.

## 6. Clinical Applications

The utilization of BoNT-A injections for orofacial pain therapy has demonstrated robust efficacy in the management of trigeminal neuralgia and neurovascular pain, and to a somewhat lesser degree for painful traumatic trigeminal neuropathy (PTTN). All these pain syndromes are chronic in nature and necessitate prophylactic treatment. The current treatment is by daily administration of medications with possible adverse effects and drug interactions, especially in older patients mostly consuming other medications. BoNT-A has the advantage of a single injection that provides a prophylactic effect for many months, and can be repeated when needed. Rare adverse effects have been reported and there are no sensory alterations in the site of injection.

As for myofascial pain the effect is less clear and there are contradictory data. However, when successful, long-term efficacy cannot be ignored. For obvious reasons, the intramuscular site was chosen for injection. However, it was reported that intramuscular injections of BoNT-A results in its axonal transport to the spinal cord via transcytosis [101], but the effect may differ for intramuscular injections in the trigeminal system. We therefore suggest that other injection sites are to be explored, which may result in better pain control for myofascial pain.

## Figures and Tables

**Table 1:** BoNT-A use in orofacial pain conditions.

Orofacial Pain Type	Efficacy Summary	Clinical Notes	Injection Sites
Trigeminal Neuralgia (TN)	BoNT-A is a viable addition to the treatment options of patients with TN. There is a need to establish proper dosing	Considered when standard treatments (e.g., carbamazepine) fail or are not tolerated; the advantage of a one-time injection is obvious	Trigger areas (e.g., gums). Intradermal or submucosal along painful trigeminal branches; avoid deep muscular injection
Post-Traumatic Trigeminal Neuropathy (PTTN)	Limited evidence; data is based on small trials and case reports, or preclinical experiments	Some patients benefit, but animal experiments demonstrated the central antinociceptive effect of BoNT-A on peripheral neuropathic pain	Near affected peripheral nerve (e.g., mental foramen region); avoid causing motor dysfunction
Myofascial Orofacial Pain (TMD/MOP)	Conflicting data with moderate to low certainty. However, long-term efficacy cannot be ignored	Some benefit in refractory cases; similar effect to anesthetics; not superior to placebo in many trials	Masseter, temporalis, medial pterygoid, and neck muscles based on trigger point’s location
Facial Migraine (Chronic Neurovascular Orofacial Pain)	A key prophylactic therapy for chronic migraine, but a lack of studies in facial migraine	Approved for chronic migraine; limited data in facial variants but rationale supported by trigeminovascular mechanisms	Forehead, scalp, or subdermal facial areas avoiding mimic muscles to prevent asymmetry

## Data Availability

The original contributions presented in this study are included in the article material. Further inquiries can be directed to the corresponding author(s).
